# The Impact of Virtual Fractional Flow Reserve and Virtual Coronary Intervention on Treatment Decisions in the Cardiac Catheter Laboratory

**DOI:** 10.1016/j.cjca.2021.06.004

**Published:** 2021-10

**Authors:** Rebecca C. Gosling, Zulfiquar Adam, David S. Barmby, Javaid Iqbal, Kenneth P. Morgan, James D. Richardson, Alexander M.K. Rothman, Patricia V. Lawford, D. Rodney Hose, Julian P. Gunn, Paul D. Morris

**Affiliations:** a Department of Infection, Immunity and Cardiovascular Disease, University of Sheffield, Sheffield, United Kingdom; b Department of Cardiology, Sheffield Teaching Hospitals National Health Service Foundation Trust, Sheffield, United Kingdom; c Insigneo Institute for In-Silico Medicine, Sheffield, United Kingdom; d Department of Circulation and Medical Imaging, Norwegian University of Science and Technology, Trondheim, Norway

## Abstract

**Background:**

Using fractional flow reserve (FFR) to guide percutaneous coronary intervention for patients with coronary artery disease (CAD) improves clinical decision making but remains underused. Virtual FFR (vFFR), computed from angiographic images, permits physiologic assessment without a pressure wire and can be extended to virtual coronary intervention (VCI) to facilitate treatment planning. This study investigated the effect of adding vFFR and VCI to angiography in patient assessment and management.

**Methods:**

Two cardiologists independently reviewed clinical data and angiograms of 50 patients undergoing invasive management of coronary syndromes, and their management plans were recorded. The vFFRs were computed and disclosed, and the cardiologists submitted revised plans. Then, using VCI, the physiologic results of various interventional strategies were shown and further revision was invited.

**Results:**

Disclosure of vFFR led to a change in strategy in 27%. VCI led to a change in stent size in 48%. Disclosure of vFFR and VCI resulted in an increase in operator confidence in their decision. Twelve cases were reviewed by 6 additional cardiologists. There was limited agreement in the management plans between cardiologists based on either angiography (kappa = 0.31) or vFFR (kappa = 0.39).

**Conclusions:**

vFFR has the potential to alter decision making, and VCI can guide stent sizing. However, variability in management strategy remains considerable between operators, even when presented with the same anatomic and physiologic data.

## Résumé

**Contexte:**

L'utilisation de la fraction du flux de réserve coronarien (FFR) pour guider une intervention coronarienne percutanée chez les patients atteints de coronaropathie améliore la prise de décision clinique mais reste sous-utilisée. La FFR virtuelle (FFRv), modélisée à partir d'images angiographiques, permet une évaluation physiologique sans capteur de pression et peut être étendue à l'angioplastie coronaire virtuelle (ACV) pour faciliter la planification de l'intervention. Cette étude a examiné l'effet de l'ajout de la FFRv et de l'ACV à l'angiographie dans l'évaluation et le soin des patients.

**Méthodes:**

Deux cardiologues ont examiné indépendamment les données cliniques et les angiogrammes de 50 patients exposés à une prise en charge invasive de syndromes coronariens, et leurs programmes de prise en charge ont été enregistrés. Les FFRv ont été calculées et divulguées, et les cardiologues ont soumis des programmes révisés. Par la suite, à l'aide de l'ACV, les données physiologiques provenant de diverses stratégies interventionnelles ont été dévoilées et une révision supplémentaire a été demandée.

**Résultats:**

La divulgation de la FFRv a conduit à un changement de stratégie dans 27 % des cas. L'ACV a conduit à un changement de la taille de l'endoprothèse dans 48 % des cas. La divulgation de la FFRv et de l'ACV a augmenté le niveau de confiance de l'intervenant dans sa décision. Douze cas ont été revus par six cardiologues supplémentaires. L'accord entre les cardiologues sur les programmes de prise en charge basés sur l'angiographie (kappa = 0,31) ou sur la FFRv (kappa = 0,39) était limité.

**Conclusions:**

La FFRv a le potentiel de modifier la prise de décision, et l'ACV peut aiguiller sur le dimensionnement de l'endoprothèse. Cependant, la variabilité de la stratégie de prise en charge reste considérable entre les opérateurs, même lorsqu'on leur présente les mêmes données anatomiques et physiologiques.

Using fractional flow reserve (FFR) to guide percutaneous coronary intervention (PCI) improves clinical outcomes and reduces costs compared with angiographic guidance.^1^ FFR also affects decisions regarding interventional strategy. In the Does Routine Pressure Wire Assessment Influence Management Strategy at Coronary Angiography for Diagnosis of Chest Pain? (RIPCORD) study, knowledge of FFR altered the recommended treatment plan in 26% of patients.^2^ However, FFR measurement is invasive, expensive, time consuming, and not available at all centres. It therefore remains underused.^3^ Computational fluid dynamics models of FFR (vFFR) based on the angiogram can predict FFR without the need for invasive instrumentation.^4^, ^5^, ^6^ Related modelling techniques also permit virtual coronary intervention (VCI), or “virtual stenting,” which enables the physiologic response to alternative stenting strategies to be predicted *a priori*.^7^ However, it remains unknown whether such virtual clinical methods have an impact on clinical decision making similar to invasive FFR.

In this study, we investigated the effect of the VIRTUheart (Medical Physics Group, Department of Cardiovascular Science, Medical School, University of Sheffield, Sheffield, UK) model of vFFR and VCI on decision making for patients with acute or chronic coronary syndromes.

## Methods

### Study design and patients

This was an observational study involving retrospective analysis of prospectively collected data from patients attending the cardiac catheter laboratory at the Northern General Hospital, Sheffield, United Kingdom, a large tertiary cardiac centre in the North of England. We interrogated the research database to identify patients who had undergone PCI for chronic or non–ST-segment elevation acute coronary syndromes (ACS). The research database has been compiled over a number of years and consists of nearly 500 coronary angiograms. These cases have already been prescreened for their suitability for coronary modelling.^7^ Seventy consecutive cases from the database, meeting the inclusion criteria, were selected for analysis. Patients were excluded if they had presented with ST-segment elevation myocardial infarction, previous coronary artery bypass graft (CABG) surgery, or chronic total coronary artery occlusions, or if the angiographic images were unsuitable for modelling. From the initial 70 patient cases, 50 were identified for inclusion in the study (in keeping with the sample size calculation). A patient flow diagram is shown in Supplemental Figure S1. The research was approved by the National Health Service Research Ethics Committee and the Institutional Review Board. Because this was an observational study using routinely collected clinical data, no formal consent was required.

### Original procedure

Patients underwent standard multiple single-plane coronary angiography before PCI. PCI was performed using standard techniques according to the operator's normal practice. Treatment decisions made by the operator at the time were noted but not disclosed to the cardiologists in this study.

### Modelling protocol

**Figure 1:**
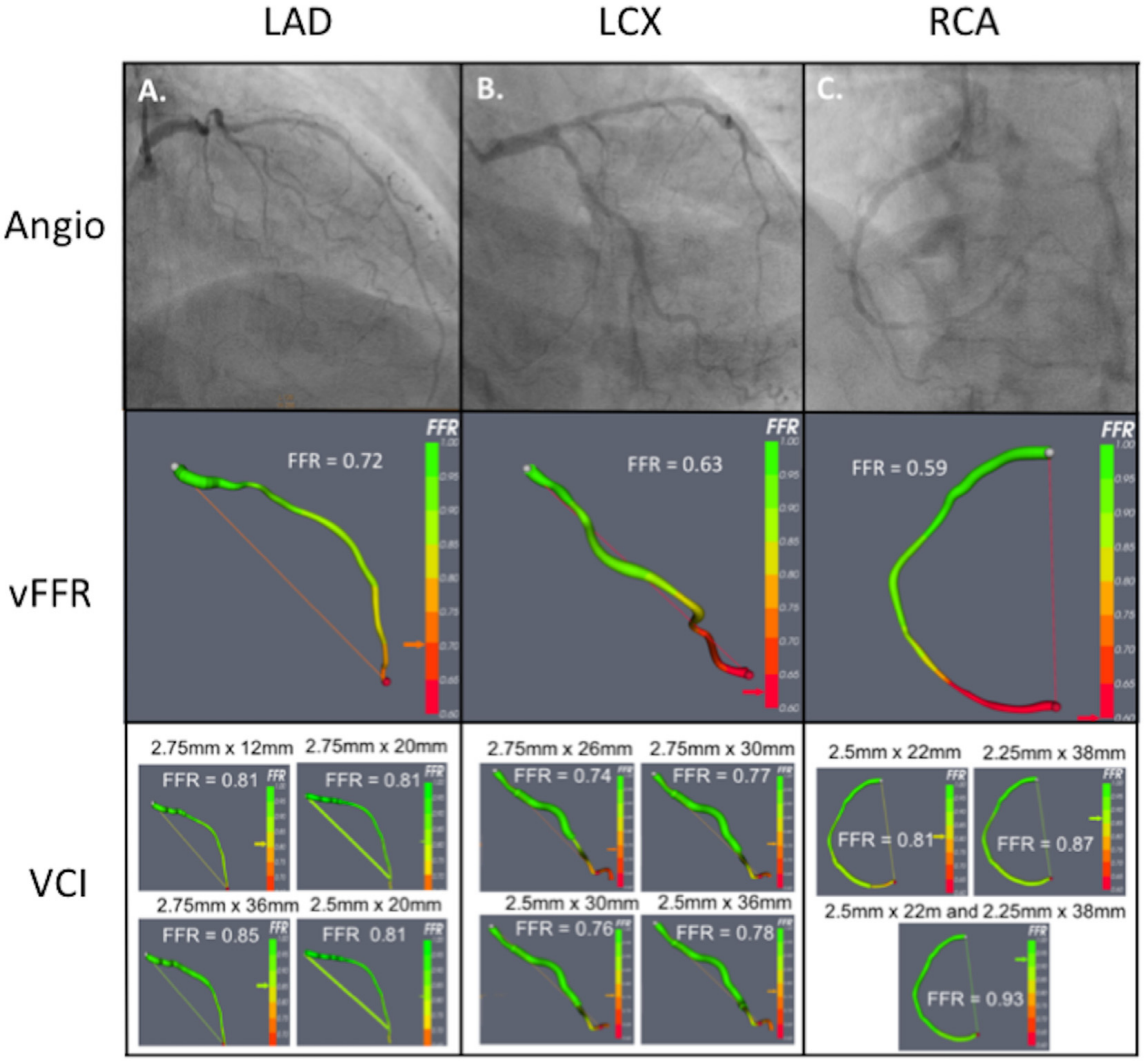
Illustrative case example. A 78-year-old woman with a background of type 2 diabetes mellitus and hypertension attended the accident and emergency department with severe chest tightness. The troponin level was > 10× the upper limit of normal. There were no localising features on electrocardiography. Baseline angiographic images of the LAD, LCX, and RCA are shown in the top left, centre, and right panels, respectively, above the corresponding vFFR and VCI results. Up to 4 VCI strategies are shown for each vessel (selected after consultation with an independent interventional cardiologist). For each, the reconstructed artery is displayed as well as the predicted post-treatment vFFR. The stent details are displayed above the image. The operators’ management plans based on angiographic, vFFR, and VCI assessment are presented in Table 1. LAD, left anterior descending artery; LCX, left circumflex artery; RCA, right coronary artery; VCI, virtual coronary intervention; vFFR, virtual fractional flow reserve.

Angiograms were screened against the criteria for accurate modelling, namely, adequate image centering, at least 2 orthogonal views, inclusion of the whole arterial segment of interest, sufficient contrast between vessel and background, minimal vessel overlap, sufficiently long acquisitions to capture several cardiac cycles with at least 1 good diastolic frame, and minimal panning. Vessels with a minimum diameter of 2.5 mm and at least 30% diameter stenosis by visual estimation were included. Cases which did not meet these criteria were excluded. With the use of the VIRTUheart system, diseased vessels were reconstructed and up to 4 alternative plausible VCI strategies were constructed, based on advice from an independent interventionist (Fig. 1 and Table 1).^8^ vFFR was computed before and after VCI.

**Table 1:** Case example: breakdown of management plans made by each cardiologist after angiographic, vFFR, and VCI assessments

	Angiographic			vFFR			VCI		
Cardiologist	Plan	Vessel(s) for PCI	Stent size	Plan	Vessel(s) for PCI	Stent size	Plan	Vessel(s) for PCI	Stent size
A	OMT		–	OMT		–	OMT		–
B	PCI	RCA	2.25 × 28 mm	PCI	RCA	2.25 × 32 mm, 2.75 × 32 mm	PCI	RCA	2.25 × 32 mm, 2.75 × 32 mm
					LCX	2.5 × 28mm		LCX	2.5 × 28mm
C	PCI	RCA	3.0 × 48 mm	PCI	RCA	3.0 × 48 mm	PCI	RCA	3.0 × 48 mm
D	PCI and PW LCX	RCA	2.5mm × 30 mm	PCI	RCA	2.5 × 38 mm	PCI	RCA	2.5 × 38 mm
E	PW LAD, if pos. surgical referral	–	–	PCI	RCA	2.75 × 30 mm	PCI	RCA	2.75 × 30 mm
F	OMT		–	OMT		–	OMT		–
G	PW LAD, if pos. surgical referral	–	–	PCI	LCX	2.5 × 23 mm	PCI	LCX	2.5 × 26 mm
H	PCI	RCA	3.0 × 38 mm	PCI	RCA	3.0 × 38 mm	PCI	RCA	3.0 × 38 mm

LAD, left anterior descending artery; LCX, left circumflex artery; OMT, optimal medical therapy; PCI, percutaneous coronary intervention; PW, pressure wire; RCA, right coronary artery; VCI, virtual coronary intervention; vFFR, virtual fractional flow reserve.

### Impact of vFFR and VCI on clinical decisions

**Figure 2:**
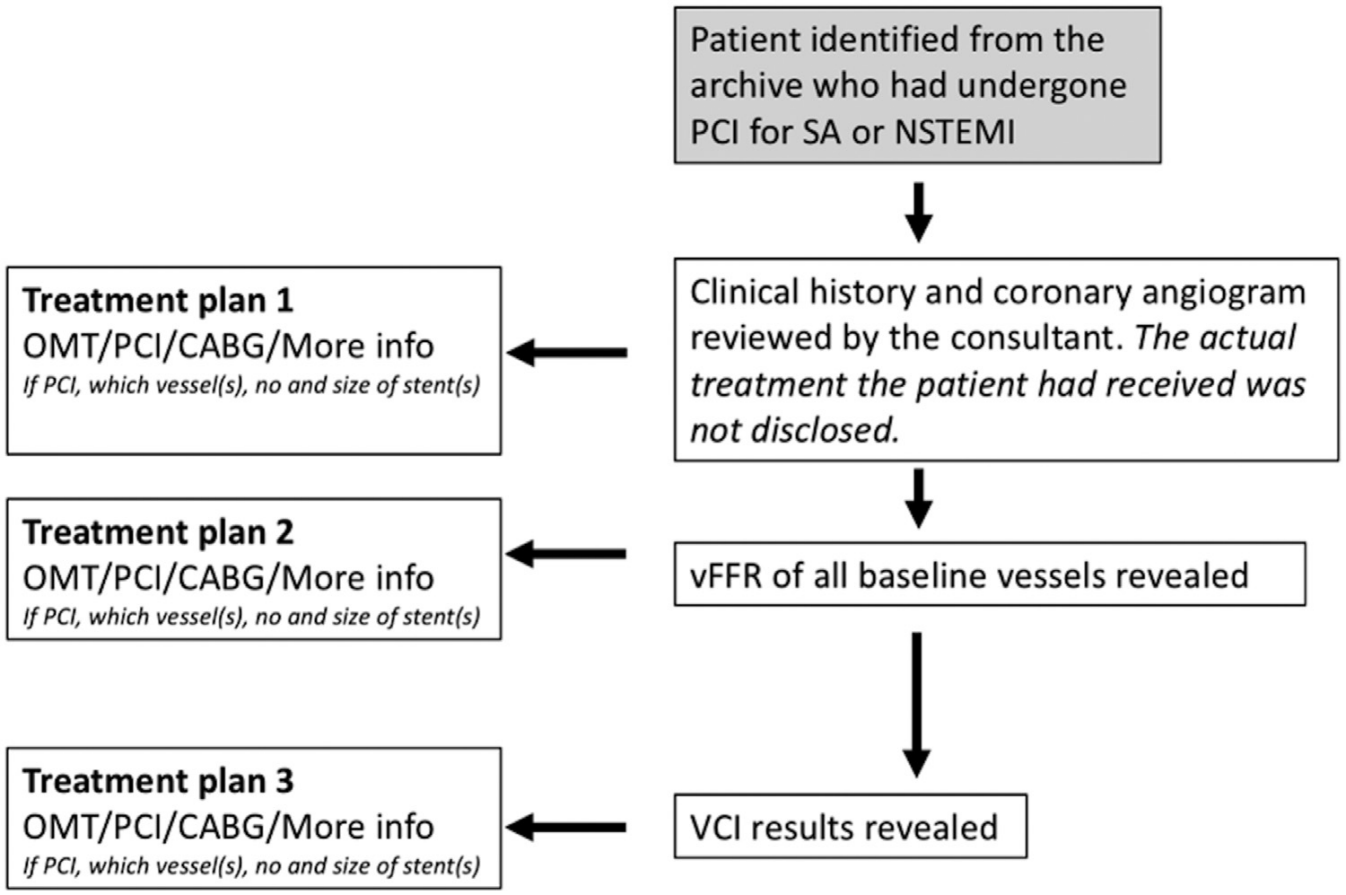
Diagrammatic representation of study protocol. CABG, coronary artery bypass graft; NSTEMI, non–ST-elevation myocardial infarction; OMT, optimal medical therapy; PCI, percutaneous coronary intervention; PW, pressure wire; RCA, right coronary artery; SA, stable angina; VCI, virtual coronary intervention; vFFR, virtual fractional flow reserve.

Cases were independently reviewed by 2 interventionists blinded to each other and to the original procedure. Each cardiologist was presented with the clinical history, electrocardiography, and baseline angiographic images. Based on these conventional data sources, they were asked to give their recommendation for treatment (on a per-patient level): optimal medical therapy (OMT), PCI, CABG surgery, or “more information required,” which could include measured FFR or any other investigation they thought was required for them to make a decision. If they selected PCI, they were asked to specify the vessel(s) for revascularisation and the number and size of stent(s) they would recommend based on their clinical practice. At each stage, they were asked to rate their confidence in their decision on a scale of 0 to 10 (10 being high). After making the initial recommendations, they were shown the results of baseline vFFR modelling (including the stent sizing tool, which displays the vessel width at any chosen point as well as the distance between any 2 prespecified points along the vessel path [Supplemental Fig. S2]) and asked to restate their management plan and their confidence in the decision based on those additional data. Finally, they were shown the VCI results and, again, were asked to state any changes in the management plan. At each stage, the interventional cardiologists were asked to utilize the vFFR and VCI data in combination with their own clinical judgment to reflect real-world practice. The importance they ascribed to the modelling was left to their discretion. All of the participating cardiologists were presented with the most recently published accuracy data for both vFFR and VCI before commencing the study.^8^ The study protocol is illustrated in Figure 2. To further explore interobserver variability, a subset of 12 cases were randomly selected and shown to 6 additional interventional cardiologists, independently from each other and the original clinical team. The cases were presented in the same way as above. The primary outcome was the number/percentage of cases in which the patient-level treatment recommendation changed based on virtual physiology.

### Analysis and sample size

Continuous data were presented as mean ± SD and categoric data as n (%) unless stated otherwise. Patient-level treatment strategies based on angiographic, vFFR, and VCI assessment were compared. Agreeability between operators was assessed with the use of Cohen's kappa coefficient. Confidence scores were compared with the use of repeated-measures analysis of variance. Statistical analysis was carried out with the use of SPSS version 21 (SPSS, New York, NY). Based on the RIPCORD study, it was estimated that a change of management would be observed in about 25% of patients; a change < 10% being deemed unimportant. The number of patients required in the study was directed by p, the proportion of cases in which the decision is different after the intervention than it was before. The 95% confidence intervals (CI) for p were derived from the following formula: p̂ ± 1.96 √ (p̂(1 − p̂)/n). A sample size of 50 provides 95% CIs of 12% to 37% for this effect size.

## Results

### Patient and vessel characteristics

Patient baseline characteristics are summarised in Table 2. Fifty potentially suitable patients were identified from hospital records, with a total of 86 diseased vessels. Eight vessels (9%) were unsuitable for vFFR modelling, so 78 lesions from 50 patients were included in the final analysis. Cases included 43 left anterior descending (LAD), 17 left circumflex (LCX), 13 right (RCA), 3 diagonal (Dx), and 2 obtuse marginal (OM) arteries. Mean baseline vFFR was 0.73 ± 0.17.

**Table 2:** Patient and lesion characteristics

Patient characteristics (n = 50)	
Age, y	66 ± 11
Male	36 (72%)
Hypertension	33 (66%)
Hyperlipidaemia	20 (40%)
T2DM	12 (24%)
Current smoker	12 (24%)
Previous MI	6 (12%)
Indication for PCI	
Stable angina	17 (34%)
NSTEMI	33 (66%)
**Vessel characteristics (n = 64)**	
Vessel	
LAD	37 (58%)
LCX	14 (22%)
RCA	10 (16%)
OM	2 (3%)
Dx	1 (2%)
Baseline vFFR	0.73 ± 0.16
No. of stents	1.1 ± 0.3
Stent length, mm	21.3 ± 7.4
Stent width, mm	3.1 ± 0.4

Values are mean ± SD or n (%).

Dx, diagonal artery; LAD, left anterior descending artery; LCX, left circumflex artery; MI, myocardial infarction; NSTEMI, non–ST-elevation myocardial infarction; OM, obtuse marginal artery; PCI, percutaneous coronary intervention; RCA, right coronary artery; T2DM, type 2 diabetes mellitus; vFFR, virtual fractional flow reserve.

### Impact of disclosing vFFR result

**Figure 3:**
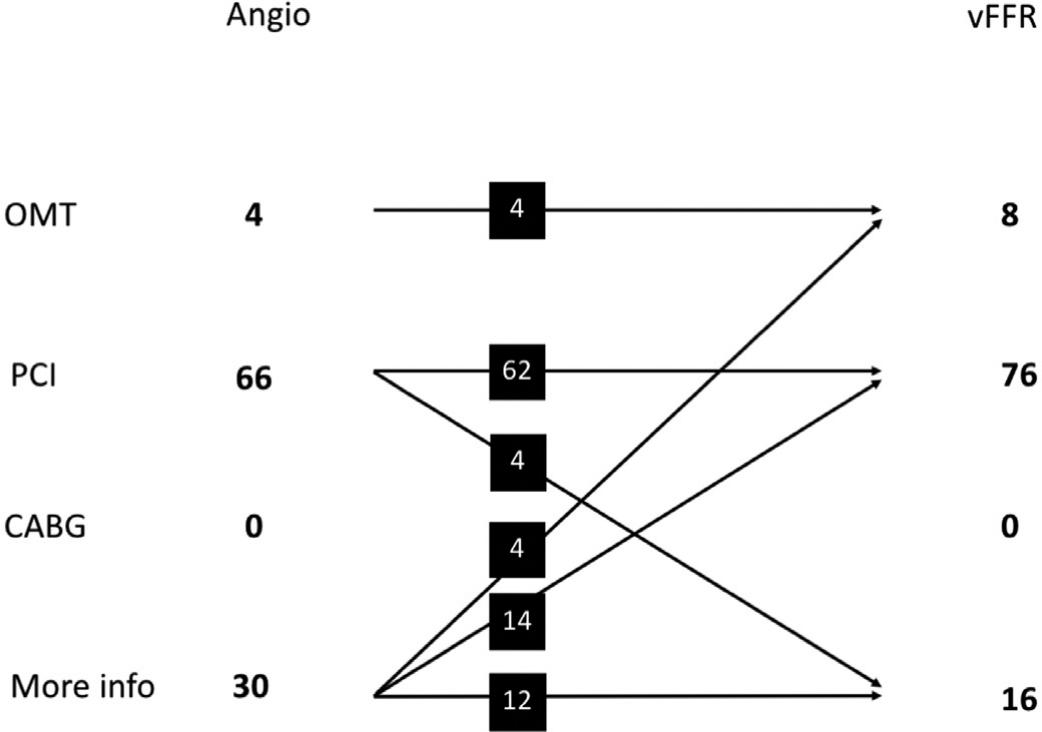
Summary of management plans made after angiographic (Angio) and virtual fractional flow reserve (vFFR) assessment. Detailed breakdown of management plan allocation by angiography alone and after vFFR assessment. CABG, coronary artery bypass graft; OMT, optimal medical therapy; PCI, percutaneous coronary intervention.

After revealing the vFFR results, the operators changed their initial management strategy on 22 occasions (22%, 95% CI 15%-31%). Each patient case was considered twice (because each case was reviewed independently by the 2 operators), so “occasion” refers to a particular case assessed by an individual operator. Details of the nature of these changes are shown in Figure 3. PCI strategy (number and location of vessels for PCI) changed in a further 5 (5%), so the total number of occasions in which management changed was 27 (27%, 95% CI 19%-36%) (20% of patients for operator A and 34% of patients for operator B). In cases where PCI was selected, vFFR resulted in a change in stent size in 47%. The amendments included an increase in length in 48%, a reduction in length in 32%, a reduction in diameter in 32%, and an increase in diameter in 10%.

### Effect of disclosing VCI results

For cases in which PCI was recommended, disclosure of the VCI results led to a change in stent size in 33% of occasions. The amendments included an increase in stent length in 44%, a reduction in stent length in 30%, a reduction in stent diameter in 22%, and an increase in stent diameter in 4%. On 1 occasion, VCI led to a change in initial strategy. This was a case with a borderline vFFR, prompting the cardiologist to recommend an invasive pressure wire. However, VCI revealed an excellent result with minimal stenting, which provided sufficient reassurance to proceed with PCI without the need for a pressure wire.

### Overall effect of vFFR and VCI

Stent size was amended with either vFFR or VCI on 48% of occasions. The amendments included an increase in length in 42%, a reduction in length in 28%, an increase in diameter in 4%, and a reduction in diameter in 25%. Mean stent widths after angiographic, vFFR, and VCI assessments were 2.91 ± 0.34, 2.85 ± 0.31, and 2.83 ± 0.32 mm, respectively (*P* = 0.04). Mean stent lengths after angiographic, vFFR, and VCI assessment were 23.0 ± 8.5, 24.2 ± 8.7 and 23.9 ± 8.3 mm, respectively (*P* = 0.37).

### Confidence in the management plan

Based on angiographic assessment alone, mean confidence scores in patient-level management, vessel-level management, and stent sizing were 8.11, 8.38, and 6.94 out of 10, respectively. Disclosure of vFFR increased operator confidence in all 3 domain: patient-level management: + 0.47 ± 1.27 (*P* < 0.001); vessel-level management: + 0.48 ± 1.23 (*P* < 0.001); stent sizing: + 1.0 ± 1.14 (*P* < 0.001). After VCI results were revealed, the confidence level in patient-level management and stent sizing both increased significantly (+ 0.14 ± 0.63 [*P* = 0.03]; + 0.72 ± 0.62 [*P* < 0.001]) but there was no significant change in confidence in vessel-level management (+ 0.07 ± 0.63 [*P* = 0.31]). The data are summarised in Table 3. Confidence in angiography-based management was not related to whether the operator went on to change their plan based on physiology or not (8.18 vs 7.82; *P* = 0.32). However, initial confidence in stent size was significantly lower in those cases in which stent size recommendation subsequently changed (6.63 vs 7.15; *P* = 0.02).

**Table 3:** Confidence scores in patient-level management, vessel-level management, and stent sizing after angiographic assessment, vFFR assessment, and VCI (scale 1-10)

	Angiographic	vFFR	VCI	*P* value
**Cardiologist A**				
Patient level	8.64 ± 1.38	8.76 ± 1.35	8.86 ± 1.31	0.04
Vessel level	9.21 ± 0.95	9.21 ± 1.01	9.25 ± 0.87	0.52
Stent size	7.34 ± 1.03	7.92 ± 0.91	8.62 ± 0.91	< 0.001
**Cardiologist B**				
Patient level	7.58 ± 1.43	8.22 ± 1.17	8.39 ± 0.92	< 0.001
Vessel level	7.59 ± 1.48	8.29 ± 1.24	8.38 ± 1.04	< 0.001
Stent size	6.56 ± 0.73	7.72 ± 0.95	8.42 ± 0.84	< 0.001
**Combined**				
Patient level	8.11 ± 1.50	8.49 ± 1.29	8.63 ± 1.15	< 0.001
Vessel level	8.38 ± 1.48	8.71 ± 1.23	8.79 ± 1.06	< 0.001
Stent size	6.94 ± 0.97	7.81 ± 0.94	8.51 ± 0.88	< 0.001

Values are mean ± SD. *P* values are for significance of change in confidence level after vFFR and VCI assessment (repeated-measures analysis of variance).

VCI, virtual coronary intervention; vFFR, virtual fractional flow reserve.

### Interobserver variability

**Figure 4:**
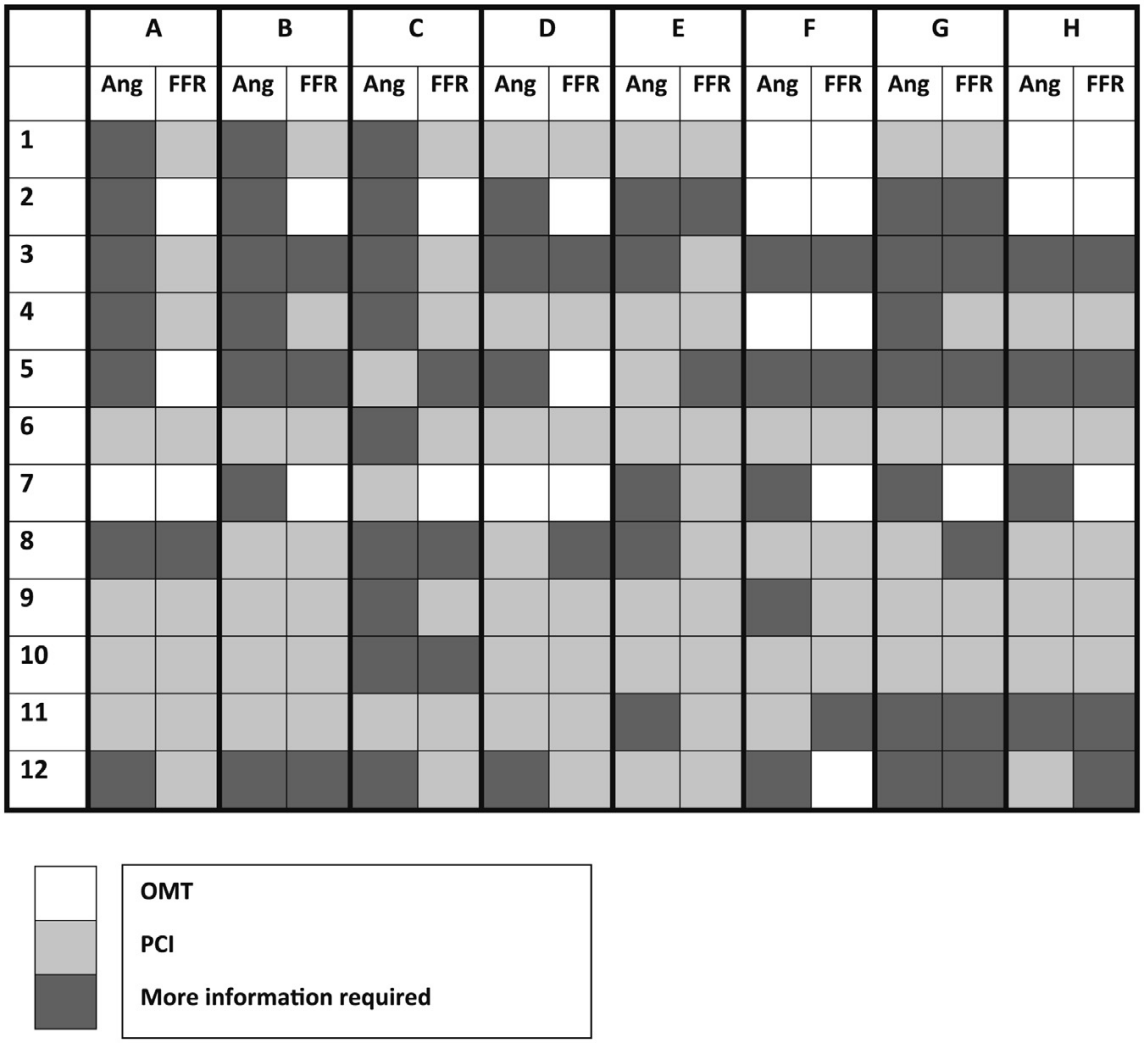
Recommended management plans provided by cardiologists for a subset of 12 patients after angiographic assessment and vFFR assessment. Twelve patient cases (**1-12**) were reviewed by 8 cardiologists (**A-H**). For each case, the cardiologist provided a management plan (OMT, PCI, CABG, or more information required) based on conventional angiography (Ang. columns). A second plan was then made after vFFR results were made available (vFFR columns). CABG, coronary artery bypass graft; OMT, optimal medical therapy; PCI, percutaneous coronary intervention; vFFR, virtual fractional flow reserve.

The subset of 12 cases reviewed independently by a total of 8 interventional cardiologists included 9 LADs, 6 LCXs, and 5 RCAs. Mean vFFR was 0.73 ± 0.15. Baseline characteristics are summarised in Table 4. There was minimal agreement between the cardiologists’ management plans either before (ie, based on the angiogram) (kappa = 0.30; 95% CI 0.21-0.39) or after (kappa = 0.39; 95% CI 0.31-0.47) vFFR assessment. All of the management plans are illustrated in Figure 4.

**Table 4:** Baseline patient and vessel characteristics for the subset of 12 patients

Patient characteristics (n = 12)	
Age, y	64 ± 10
Male	8(67%)
Hypertension	7 (58%)
Hyperlipidemia	5 (42%)
T2DM	1 (8%)
Current smoker	4 (33%)
Previous MI	2 (17%)
Indication for PCI:	
Stable angina	4 (33%)
NSTEMI	8 (67%)
Vessel characteristics (n = 20)	
Vessel	
LAD	9 (45%)
LCX	6 (30%)
RCA	5 (25%)
Baseline vFFR	0.73 ± 0.15

Values are mean ± SD or n (%).

LAD, left anterior descending artery; LCX, left circumflex artery; MI, myocardial infarction; NSTEMI, non–ST-elevation myocardial infarction; PCI, percutaneous coronary intervention; RCA, right coronary artery; T2DM, type 2 diabetes mellitus; vFFR, virtual fractional flow reserve.

## Discussion

In this study, we have analysed the potential for angiography-based computed coronary physiology, namely, vFFR with its derivative, VCI, to alter patient management. Knowledge of the baseline vFFR led to a change in management in 27%. VCI led to a change in recommended stent size in 48%. Of note, the proportion of cases in which management was changed based on the physiology varied greatly, and when 8 cardiologists were studied, the proportion of patients in whom changes were recommended varied from none to 50% (average 33%). There were also marked differences among their management plans. However, both vFFR and VCI significantly improved the cardiologist's confidence in their management plans.

### Impact of vFFR on patient management

When baseline vFFR results were revealed, a change in the proposed management plan occurred in 27%-33% of patients. The effect of revealing coronary physiology on cardiologists’ decision making has previously been examined in the RIPCORD^2^ and Does the Routine Availability of CT-Derived FF Influence Management of Patients With Stable Chest Pain Comapred to CT Angiography Alone? (FFR_CT_ RIPCORD)^9^ trials. In RIPCORD, there was a change in the patient-specific management plan in 26% of cases with FFR compared to angiography alone, and in the **F**ractional Flow Reserve vs **A**ngiography in Guiding **M**anagement to **O**ptimise O**u**tcome**s** in **N**on-**ST**-Segment **E**levation**M**yocardial **I**nfarction (FAMOUS-NSTEMI) trial it was 22%,^10^ remarkably similar to the proportions in our study. Our study differed from RIPCORD and FAMOUS-NSTEMI in a number of ways. First, ours included both chronic and acute coronary syndromes, reflecting current practice.^11^ Second, only patients who had initially been selected for PCI were included. This was to ensure that there was a high proportion of lesions to assess. Third, and most importantly, RIPCORD and FAMOUS-NSTEMI used invasive FFR, whereas our study used vFFR which is not yet as well validated as invasive FFR.^12^^,^^13^ Fourth, in our study, to explore the impact of virtual coronary physiology in real world practice, the interventional cardiologist was asked to incorporate the vFFR and VCI data into their management plan as they saw fit, based on the whole clinical, angiographic, and physiologic setting, without mandating treatment based solely on the vFFR. This probably explains the wide variation in treatment recommendations between individual cardiologists when presented with the same vFFR. In acute cases, we found that operators frequently chose to proceed to revascularisation regardless of the vFFR. In the FFR_CT_ RIPCORD study, FFR_CT_ changed treatment decisions compared with those made based on angiography alone in 36% of cases.^9^ The single largest group change was from “more information required” (ie, an invasive pressure wire) to either OMT or PCI, constituting 53% of the cases in which management changed. This accorded with our findings (70%). In our study, an invasive pressure wire was recommended in 30% of cases. Although this is higher than the observed usage of 5%-10%,^3^ because this was a virtual study this might not translate into actual pressure wire usage; in the FFR_CT_ RIPCORD study, the equivalent figure was 19%. Moreover, this study was carried out in a tertiary cardiology centre with ready access to pressure wire usage.

### Interobserver variability

Our initial findings of a large variation in recommendations between our two experts mandated further study with a larger group of interventional cardiologists. When the same patient cases were reviewed by 8 cardiologists, patient-level management changed based on vFFR in 33%, but the range was 0%-50%—thus, the impact of vFFR was considerable, but the difference between operators was even greater. There was also significant variation between management plans, with minimal increase in agreement following vFFR disclosure. Interobserver variability in assessing coronary angiograms is well documented,^14^, ^15^, ^16^, ^17^, ^18^ but the impact upon treatment decisions is less well known. In our study, a major factor was trust in the vFFR, especially when the 3-dimensional (3D) reconstruction differed from the cardiologist's perception of the angiogram. Despite several studies demonstrating disagreement between visual and physiologic assessment, many operators consider angiography to be superior. The Evolving Routine Standards of FFR Use (ERIS) study^19^ analysed the use of physiologic assessment in 76 centres. Invasive physiology was used in fewer cases than recommended, the predominant reason being confidence in the history and the angiogram. We found that the operators’ initial confidence in their management plan was unrelated to their decision according with physiology or whether they went on to change their plan based on physiology. This suggests that being confident in angiographic assessment is not a good reason to refrain from physiologic assessment. In our study, in an average of 38%, after vFFR was made available the management plan still contradicted what would be recommended by vFFR alone. The most common reason for this (33%) was the presence of other clinical or technical factors that precluded PCI, such as diffuse disease, distal disease, or noninvasive imaging confirming nonviability. However, in 22% of cases, the operator stated that they were more convinced by their angiographic assessment than by the vFFR.

### Impact of VCI on treatment planning

Although disclosure of the VCI results had little impact on patient-level management beyond that achieved with vFFR, the procedural details (size of stent) changed in 33% of cases based on VCI alone, and in 48% when combined with the stent sizing feature. VCI is intended to be a treatment planning tool, so its main use is in cases in which the operator has already decided that PCI is warranted, based on either angiographic or physiologic assessment. VCI then allows the operator to plan the procedure more precisely. We demonstrated, for the first time, that this approach has the potential to significantly affect treatment decisions. This could maximise physiologic benefit from PCI, potentially leading to improved outcomes, and possibly reduce the risks of over- or undersizing and excessive stent length. This concept needs to be explored. In addition, vFFR allied with VCI may offer the noninterventional cardiologist appreciation of the possibilities for treatment. Previous work demonstrates that VCI based on invasive pressure wire data is not only more accurate but can also generate absolute flow and microvascular data.^20^

### Clinical applicability in the future

For the purpose of this study, vFFR and VCI were performed in all cases regardless of complexity. In reality, not all cases would require vFFR and/or VCI, and determining when they should be used remains an important question. A severe lesion or a completely normal vessel does not warrant vFFR. Its benefit, like invasive FFR, is in moderate lesions where the hemodynamic significance is unclear. However, correctly identifying these cases remains challenging. The purpose of VCI is for treatment planning, so it is most relevant in cases where the operator is unsure on the optimal stenting strategy regardless of the baseline vFFR (eg, 1 vs multiple stents in the setting of diffuse or tandem lesions). Ultimately it will be up to the operator when they wish to use these technologies, so more work is required to provide outcome data and convince cardiologists that a virtual physiology–based approach is superior to an angiography-based approach. Significant variation in the confidence in the virtual technology when it disagreed with the operator's angiographic assessment was a key contributor to the interobserver variability demonstrated in this study.

### Limitations

First, only patients undergoing PCI were studied. We could not assess the potential impact on a wider group of patients with coronary disease. Second, the sample was relatively small. Third, stent sizing decisions were made without the aid of balloon markers, intravascular imaging, or other cues which would normally be available to assist the operator with sizing during an invasive procedure. Fourth, vFFR was computed with the use of generic boundary conditions, although previous work has demonstrated acceptable accuracy with this method. All operators were advised of the accuracy of the tools before they began their assessment. Fifth, in a virtual study with modest numbers, we cannot report on complications or outcomes. Sixth, operators were encouraged to state their treatment recommendations based on real-life practice, but because this was a virtual study, it was not possible to control for potential bias. Seventh, this was not an all-comers study; cases were selected from a prescreened research database. We have previously reported that the proportion of “real-world” cases that are suitable for coronary modelling is about 80%.^7^ Eighth, our cases include a higher proportion of LAD arteries than LCX and RCA owing to a slightly higher exclusion rate of these arteries because of difficulties with the 3D reconstruction. The LAD is generally well imaged in multiple views and its course tends to be less torturous, which permits more accurate segmentation (3D reconstruction). The RCA is more challenging as it typically traverses multiple planes, which makes the selection of truly orthogonal views more challenging. Moreover, often only 2 images of the RCA are routinely acquired, so there are no alternative images available if one is unsuitable. However, the software is continually being updated to overcome these issues. A larger study would be required to determine the true magnitude of this effect.

## Conclusion

Disclosure of vFFR can lead to a change in planned patient management in about a third of cases compared with angiography-based assessment. Combining our novel stent sizing tool with VCI resulted in change in recommended stent sizing in almost half. Virtual physiology and VCI increased operator confidence in their selected treatment strategy. However, the treatment plans, and how virtual physiology was incorporated into them, varied significantly between interventional cardiologists. Our findings suggest that virtual physiology has the potential to alter management; but, as with measured indices, it remains the interventional cardiologist who places this into the context of the clinical picture, and their own decision making algorithms, with varying results.

## Funding Sources

Dr Gosling was supported by a British Heart Foundation Clinical Research Training Fellowship (FS/16/48/32306) and Dr Morris by a Wellcome Trust Clinical Research Career Development Fellowship (214567/Z/18/Z).

## Disclosures

The authors have no conflicts of interest to disclose.

## Appendix Supplementary materials

Image, application 1
