# Supplementary material for: The Impact of Virtual Fractional Flow Reserve and Virtual Coronary Intervention on Treatment Decisions in the Cardiac Catheter Laboratory
Source: Can J Cardiol. 2021 Oct;37(10):1530–8. doi: 10.1016/j.cjca.2021.06.004 (PMC8639608; doi:10.1016/j.cjca.2021.06.004)

Supplemental Figure S1: Patient flow diagram

Seventy patients who had undergone PCI for chronic or non ST segment elevation ACS were identified from the research database and screened for inclusion in the study. Twenty patients were excluded (seven had previous CABG surgery, 4 had chronic total occlusions, 9 had no ECG trace). A further 8 vessels were excluded as the images were not suitable for modelling.

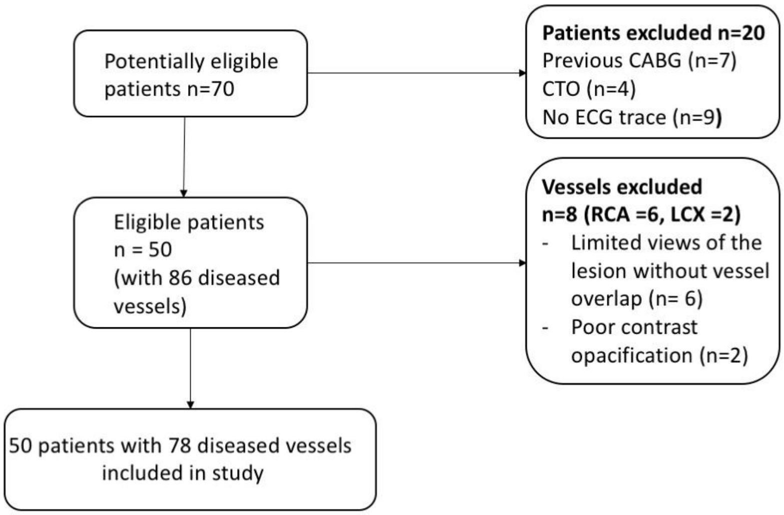

Supplemental Figure S2: The VirtuHeart vFFR and VCI interface

For vFFR (top panel), the vessel reconstruction is displayed along with the corresponding diameter data. The grey and red dots on the reconstruction can be moved along the vessel allowing the dimensions at any chosen point to be checked. The length between the two points is also displayed. The angiographic images from which the reconstruction was created are displayed in the bottom left of the panel. For VCI (bottom left), the 3-dimensional reconstruction of the artery is displayed on the screen, and the operator marks the arterial location where they wish to deploy a stent identified by the red (proximal) and blue (distal) markers. In the text (left)**,** the vessel radii at both selected points are displayed as well as the distance between t hem. The operator can adjust the radius of the desired virtual stent in the box below (“stent size”)**.** The length can be altered by moving the position of the red and blue dots. In the example shown, a 3.0mm × 20mm virtual stent has been inserted by the operator. The surface mesh is manipulated to match these stenting criteria and the new vFFR calculated (bottom right).

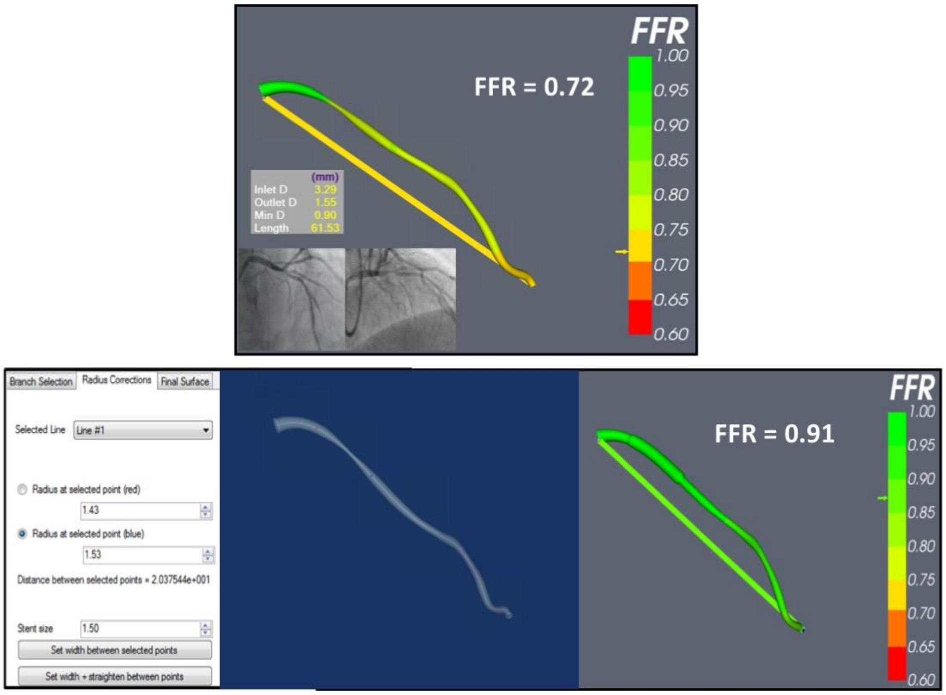

Supplement: Supplementary file 1 [file mmc1.pdf]
